# Feasibility of the Virtual Reality-based Anxiety Behavior Evaluation System (VRABES) for patients with panic disorder.

**DOI:** 10.1192/j.eurpsy.2023.458

**Published:** 2023-07-19

**Authors:** J. Kim, S.-H. Kim

**Affiliations:** Department of Psychiatry, Korea University Guro Hospital, Seoul, Korea, Republic Of

## Abstract

**Introduction:**

The high recurrence rate and diagnostic stability are current problems in treating panic disorder. Because anxiety symptoms are often temporary, it is hard to evaluate anxiety behaviors objectively. In evaluating anxiety behavior, virtual reality is suitable tools that can help bridge the gap between where the symptoms are and where the treatment is given.

**Objectives:**

This study aims to develop VRABES, an anxiety behavior evaluation system for objectively assessing an individual’s anxious behavior, and to evaluate the feasibility of VRABES.

**Methods:**

Patients with panic disorder (ANX group) and healthy controls (CON group) matched for sex, age, and marital status were recruited through outpatient clinics and public advertisements. VRABES consists of four modules; Baseline evaluation (module 0), Daily environment exposure (module 1), Relaxation (module 2), and Interoceptive exposure (module 3). Except for the Baseline evaluation module, the other three modules consisted of three steps, including 1) pre-evaluation, 2) virtual environment 1, and 3) virtual environment 2. In VRABES, subjective anxiety experience (AS) were collected for three times (pre, during, post) for module 1, 2, and 3. we conducted a repeated-measures analysis of covariance (ANCOVA) to explore any significant differences in self-rating anxiety scores among groups and repetition for each module controlling for age, sex, smoking usage, alcohol usage, and depression. Additionally, partial correlation coefficients were calculated on the relationships between measures in VRABES and Panic disorder Severity Scale (PDSS) in the ANX group to eliminate the effects of demographic variables (age, sex, smoking usage, alcohol usage), and other psychological assessment scores [Liebowitz Social Anxiety Scale: Self-Report Version (LSAS-SR), Generalized Anxiety Disorder Scale (GAD-7), and Hospital Anxiety and Depression Scale (HADS)].

**Results:**

Table presents the significant results of repeated-meausre ANCOVA. Figure shows the significant results among the paired t-tests for each group conducted as a post-hoc test for the interaction effect shown in Module 1 and Module 2.

**Table.** Results of repeated-measured ANCOVA for self-rating anxiety scores in the two groups (ANX and CON) and different time (pre, during, and post) concerning each module.

variable	Main effect-group	*post-hoc*	Main effect-time	*post-hoc*	Interaction effect	*post-hoc*			
	*F*	*p-value*		*F*	*p-value*		*F*	*p-value*	
Module1	**11.373**	**0.002**	**CON < ANX**	**4.239**	**0.017**	**pre < post during < post**	**4.085**	**0.02**	**see Fig**
Module2	**6.736**	**0.013**	**CON < ANX**	0.474	0.624		**4.198**	**0.018**	**see Fig**
Module3	**5.24**	**0.027**	**CON < ANX**	0.225	0.799		0.061	0.941

There are no significant results found in partial correlation analysis between PDSS scores and self-rating anxiety scores from VRABES.

**Conclusions:**

The results showed that the VRABES is a reliable and valid research tool.

**Disclosure of Interest:**

None Declared

